# Dietary diversity, migration experience, and brain volume in middle-aged and older adults in rural Japan: a cross-sectional magnetic resonance imaging study

**DOI:** 10.3389/fpubh.2026.1810346

**Published:** 2026-06-26

**Authors:** Hisashi Takahashi, Fumitoshi Niwa, Toshiya Ochiai, Satoshi Teramukai, Tomoyuki Ohara, Toshiki Mizuno, Masanori Nakagawa

**Affiliations:** 1 Department of Neurology, Kyoto Prefectural University of Medicine, Kyoto, Japan; 2 Department of Neurology, North Medical Center, Kyoto Prefectural University of Medicine, Yosano, Japan; 3 Department of General Medicine & Community Healthcare, Kyoto Prefectural University of Medicine, Kyoto, Japan; 4 Department of Surgery, North Medical Center, Kyoto Prefectural University of Medicine, Yosano, Japan; 5 Department of Biostatistics, Graduate School of Medical Science, Kyoto Prefectural University of Medicine, Kyoto, Japan

**Keywords:** aging, brain atrophy, lifestyle factors, neuroimaging, SIENAX

## Abstract

**Objective:**

To identify lifestyle, social, and health-related factors associated with normalized brain volume (NBV), an imaging marker of brain atrophy, among community-dwelling middle-aged and older adults in a rural Japanese population.

**Methods:**

This cross-sectional magnetic resonance imaging (MRI) study was conducted within the Tango Iki-iki (Actively Living) Longevity Study, a longitudinal community-based cohort in rural Kyoto, Japan. Participants who underwent brain MRI following cognitive screening were included. NBV was measured from T1-weighted MRI using SIENAX. Associations between NBV and demographic, lifestyle, social, and health-related factors were evaluated using linear regression analyses. Variables associated with NBV after adjustment for age and sex were further examined in multivariable models. Sensitivity analyses and principal component analysis (PCA) of dietary intake patterns were also performed.

**Results:**

A total of 235 participants were included in the MRI analysis. After adjustment for age and sex, higher dietary diversity score (DDS) (*β* = 925 mm^3^ per point; 95% CI, 176–1,674; *p* = 0.016) and migration experience (*β* = 18,294 mm^3^; 95% CI, 226–36,363; *p* = 0.048) were significantly associated with greater NBV. In the multivariable model including age, sex, DDS, and migration status, both DDS (*β* = 865 mm^3^ per point; 95% CI, 101–1,629; *p* = 0.028) and migration experience (*β* = 20,653 mm^3^; 95% CI, 443–40,862; *p* = 0.046) remained independently associated with greater NBV. These findings were generally consistent across sensitivity analyses, including a fully adjusted model. PCA suggested that vegetables, fruits, seaweed, mushrooms, and potatoes contributed strongly to dietary diversity. Additional analyses indicated that higher DDS was associated with educational attainment, cohabitation status, and lower smoking exposure in the overall cohort.

**Conclusion:**

Higher dietary diversity and migration experience were independently associated with greater NBV among middle-aged and older adults in a rural Japanese population.

## Introduction

Various behavioral, social, and health-related factors have been reported to influence brain aging and dementia risk (1). These include modifiable factors such as diet and smoking, as well as longer-term influences such as residential environment and educational attainment. Identifying these health-related factors within specific regional populations enables the development of more tailored and effective public health policies. Further, integrating and comparing results from diverse regional cohorts may lead to a more comprehensive and generalizable understanding of how lifestyle and social determinants contribute to brain aging.

In this context, we initiated a longitudinal cohort study in 2014 in the Tango region of Japan, named the “Tango Iki-iki (Actively Living) Longevity Study” (2, 3). This area is located in the northern part of Kyoto Prefecture, extending over an area approximately 70 kilometers in diameter. The region includes small urban centers, fishing villages, and mountainous communities, and is characterized by advanced aging and depopulation, providing a representative model of an aging rural society.

This study was designed to investigate aging-related diseases, particularly dementia, and to identify relevant risk and protective factors within this unique local population. To detect aging-related changes before the onset of overt functional decline, participants were enrolled at baseline ages of approximately 60–65 years. Participants were longitudinally evaluated for their living conditions, physical characteristics, motor function, and cognitive performance. During follow-up, participants suspected of cognitive decline underwent brain magnetic resonance imaging (MRI) to assess structural changes. Previous studies have shown that structural changes in the brain often precede the clinical onset of dementia, and total brain volume has been recognized as an early indicator of neurodegeneration and a predictive marker of dementia risk (4–6). In the present study, we analyzed normalized brain volume (NBV) derived using SIENAX, which estimates brain parenchymal volume and applies a scaling normalization based on skull size (7). By accounting for individual differences in head size, NBV reflects relative brain tissue volume rather than absolute brain size. Because skull size remains stable in adulthood, NBV can be interpreted as an indicator of brain atrophy.

The present study aimed to comprehensively investigate factors associated with NBV among community-dwelling middle-aged and older adults in the rural Japanese population. Given the limited evidence available from rural populations, this study adopted an exploratory approach to identify factors potentially associated with brain volume and to generate hypotheses for future research. By examining these multidimensional influences within the well-characterized cohort, we sought to clarify region-specific determinants of brain volume and to provide a foundation for future strategies to promote cognitive health and healthy aging.

## Materials and methods

### Study design and participants of Tango Iki-iki longevity cohort

This longitudinal, community-based observational study was conducted among older adults residing in Miyazu City, Kyotango City, Yosano Town, and Ine Town in the Tango region of northern Kyoto Prefecture, Japan.

**Figure 1:**
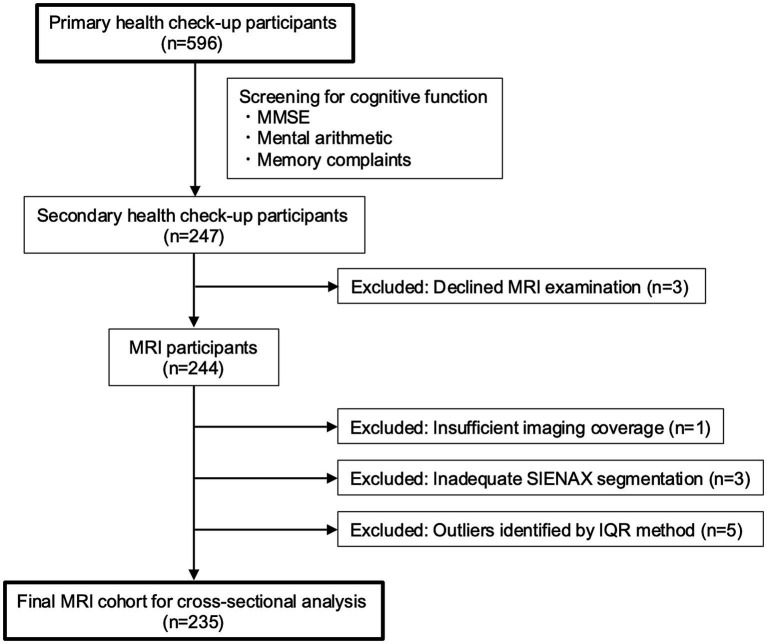
Flowchart of participant selection and study design. Participants were enrolled through primary health check-ups, and selected for cross-sectional magnetic resonance imaging (MRI) analyses after screening and exclusion. IQR, interquartile range, MMSE, mini-mental state examination.

Baseline examinations began in October 2014 for residents aged 59–66 years and were repeated approximately every 2 years. Data included in the present analysis were collected through March 2025. Each examination included lifestyle questionnaires and cognitive screening assessments. Based on the initial cognitive screening results, participants in whom mild cognitive impairment was suspected were invited to undergo secondary health check-ups, including Clinical Dementia Rating (CDR) assessment when feasible, and brain MRI. The screening assessment included mini-mental state estimation (MMSE) items (particularly orientation and delayed recall), delayed recall of a name-and-address phrase, subjective memory complaints, family-reported forgetfulness, and a simple mental arithmetic task. Participants meeting at least one of these criteria were referred for further evaluation. A flowchart of study design and participant inclusion is presented in Figure 1.

### MRI acquisition and data selection

#### MRI protocol

All brain MRI was performed using the same 3.0-T Magnetom Skyra scanner (Siemens Healthineers, Germany) with a 32-channel head coil (North Medical Center, Kyoto Prefectural University of Medicine). T1-weighted and two-dimensional fluid-attenuated inversion recovery images were obtained using a standardized protocol: repetition time = 2,100 ms; echo time = 12 ms; inversion time = 895 ms; flip angle = 130°; field of view = 220 mm; matrix = 320 × 175; slice thickness = 4 mm; and voxel size = 0.7 × 0.7 × 4.0 mm^3^.

#### Brain volume analysis

The NBV was calculated from the first MRI scan obtained at the initial secondary check-up using SIENAX (part of FSL) (7). SIENAX estimates brain parenchymal volume and applies a scaling normalization based on skull size to account for individual differences in head size. The NBV output from SIENAX was used for subsequent analyses. All SIENAX analyses were conducted using the default parameters. Visual quality control was performed for all segmentation results, and cases with obvious segmentation failures were excluded from the analysis.

#### Outlier identification

Outliers were excluded using a two-step procedure:

1. Visual inspection: Poor-quality scans or implausible volume estimates were removed;
2. Statistical outliers: Identified using the interquartile range (IQR) method, defined as values below Q1 – [1.5 × IQR] or above Q3 + [1.5 × IQR].

### Outcome measures and explanatory variables

The primary outcome of this MRI study was NBV. The following lifestyle and demographic factors were examined as explanatory variables:

- Medical history: hypertension, diabetes, and Hyperlipidemia (regardless of current treatment status);
- Smoking status: presence or absence of smoking history, current smoking status, and Brinkman index;
- Alcohol consumption: history of alcohol use and current drinking frequency (less than once per week, at least once per week, or daily);
- Dietary diversity: assessed using dietary diversity scores (DDS);
- Oral health: number of remaining teeth (self-reported);
- Sociodemographic characteristics: marital status (currently married or not), cohabitation status (currently living with others or not), and educational attainment (junior high school, high school, or ≥ vocational school, college or university);
- Migration experience: participants born outside the Tango region, regardless of the timing of relocation (migration experience), versus participants born within the Tango region (no migration experience).

To evaluate dietary diversity, DDS was calculated as the sum of 49 individual food items. Each item was scored based on the self-reported frequency of intake, as:

- 0 = less than once per week
- 1 = once or more per week
- 2 = daily consumption

Thus, DDS ranged from 0 to 98. Unless otherwise noted, participants with missing values for individual food items were excluded from DDS-related analyses.

### Statistical analysis

All analyses were performed using R (version 4.2.0) and Python (version 3.10.4). Linear regression analyses were used to evaluate associations between NBV and candidate variables. Unadjusted linear regression analyses were first performed to explore associations between NBV and each candidate variable. Subsequently, age- and sex-adjusted linear regression analyses were performed for each lifestyle and sociodemographic factor. Variables showing significant associations after age and sex adjustment were then included in a multivariable linear regression model to evaluate their independent associations with NBV. To account for potential heteroscedasticity, Heteroscedasticity-consistent robust standard errors (HC3) were used for all regression analyses. Sensitivity analyses were conducted by treating missing dietary responses as zero intake, by using conventional ordinary least squares standard errors in place of HC3 robust standard errors, and by fitting a fully adjusted model including demographic, social, and clinical factors. Values of *p* < 0.05 were considered statistically significant.

To explore dietary patterns and identify which food items contributed most strongly to dietary diversity, we conducted principal component analysis (PCA) using the intake frequencies of 49 food items. All variables were standardized prior to analysis. Factor loadings were examined to characterize dietary patterns, with higher loadings indicating greater contributions of individual food items to each principal component.

Additional exploratory analyses were conducted to identify factors associated with DDS and migration experience. Age- and sex-adjusted linear regression models were used for DDS, whereas age- and sex-adjusted logistic regression models were used for migration status.

### Ethical considerations

This study was approved by the Ethics Committee of Kyoto Prefectural University of Medicine (approval no. ERB-G − 21). All participants provided written informed consent prior to participation. All data were anonymized before analysis, and the study adhered to the principles of the Declaration of Helsinki.

## Results

### Study participants

A total of 596 participants underwent the primary health check-up of the Tango Iki-iki Longevity Study (Figure 1). Based on cognitive screening, 247 individuals were referred for a secondary assessment. Of these, 244 underwent brain MRI. One participant was excluded because of insufficient imaging coverage. Visual inspection of the SIENAX outputs identified three cases with clearly inadequate brain segmentation, which were therefore excluded. Among the remaining 240 participants, five were identified as outliers, resulting in a final analytical sample of 235 participants.

At the time of the MRI examination, no participants had been diagnosed with dementia. Among participants who underwent MRI analysis and completed CDR assessment, all were classified as CDR 0 or 0.5, with no participants classified as CDR 1 or higher. Participant characteristics for the MRI study group (*n* = 235) and the entire cohort (*n* = 596) are presented in Table 1.

**Table 1:** Characteristics of the MRI study and entire cohort participants.

Variable	MRI cohort	Entire cohort
Age (years)	62.8 ± 2.4 (*n* = 235)	61.4 ± 1.6 (*n* = 596)
Female	126/235 (53.6%)	366/596 (61.4%)
Body mass index (kg/m^2^)	22.8 ± 3.4 (*n* = 235)	22.8 ± 3.4 (*n* = 596)
Mini-Mental State Examination score	28.0 ± 2.0 (*n* = 235)	28.6 ± 1.8 (*n* = 595)
Diabetes	19/235 (8.1%)	43/596 (7.2%)
Hypertension	71/235 (30.2%)	163/594 (27.4%)
Hyperlipidemia	54/235 (30.0%)	135/595 (22.7%)
Number of remaining teeth	21.4 ± 7.5 (*n* = 233)	22.1 ± 7.1 (*n* = 583)
Current smoker	22/235 (9.4%)	44/596 (7.4%)
Smoking history	109/235 (46.4%)	230/596 (38.5%)
Brinkman index	251.1 ± 414.9 (*n* = 230)	208.4 ± 411.7 (*n* = 587)
Alcohol history	140/234 (59.8%)	344/596 (57.7%)
Frequency of current drinking ≥ daily	48/229 (21.0%)	144/596 (24.1%)
Dietary diversity score	31.5 ± 10.2 (*n* = 210)	30.8 ± 9.0 (*n* = 533)
Education ≥ college	72/211 (34.1%)	195/538 (36.2%)
Cohabiting	208/235 (88.5%)	544/595 (91.2%)
Currently married	174/215 (80.9%)	474/564 (84.0%)
Migration	48/235 (20.4%)	118/596 (19.8%)
Clinical dementia rating = 0	63/109 (57.8%)	NA
Normalized brain volume (×10^3^ mm^3^)	1421.9 ± 57.8	NA

Values are presented as mean ± standard deviation or *n*/*N* (%). Lifestyle and sociodemographic variables for the MRI cohort were obtained from the most recent health check-up preceding MRI acquisition, whereas those for the entire cohort were obtained from the baseline health check-up. NA = not applicable.

### Brain volume and its association with dietary diversity and migration

The cross-sectional MRI analysis included all 235 participants for whom structural brain images and lifestyle data were available. Mean NBV was 1.42 × 10^6^ mm^3^ (SD = 57,570), and the data showed an approximately normal distribution (Supplementary Figure S1). At first, we performed unadjusted linear regression analyses between NBV and each variable (Supplementary Table S1). Age, sex, dietary diversity, smoking-related variables, alcohol-related variables, migration status, and number of remaining teeth showed associations with NBV at a significance level of *p* < 0.05.

To identify candidate factors associated with brain volume, we then performed age- and sex-adjusted linear regression analyses between NBV and each lifestyle and sociodemographic variable (Table 2). In these analyses, higher dietary diversity (*β* = 925 mm^3^ per DDS point, 95% confidence interval [CI] 176–1,674; *p* = 0.016) and migration experience (yes vs. no; *β* = 18,294 mm^3^, 95% CI 226–36,363; *p* = 0.048) were significantly associated with greater NBV.

**Table 2:** Age- and sex-adjusted linear regression results for normalized brain volume.

Variable	*N*	*β* (95% CI)	*p*-value
Dietary diversity score	210	925 (176 to 1,674)	**0.016**
Migration	235	18,294 (226 to 36,363)	**0.048**
Cohabitant	235	7,000 (−549 to 14,549)	0.07
Mini-Mental State Examination score	235	−2,658 (−6,509 to 1,193)	0.177
Diabetes	235	−17,020 (−39,470 to 5,429)	0.139
Number of remaining teeth	233	597 (−421 to 1,615)	0.252
Smoking history	235	−10,356 (−33,739 to 13,026)	0.386
Brinkman index	230	−9.4 (−31.5 to 12.7)	0.405
Hyperlipidemia	235	7,061 (−9,953 to 24,074)	0.416
Body mass index	235	886 (−1,278 to 3,050)	0.423
Currently married	215	−6,891 (−26,018 to 12,237)	0.481
Education	211	3,172 (−9,206 to 15,551)	0.616
Current smoker	235	−6,871 (−35,028 to 21,287)	0.633
Alcohol history	234	−3,220 (−20,059 to 13,619)	0.708
Frequency of current drinking	229	−1,596 (−11,978 to 8,787)	0.764
Hypertension	235	−1,835 (−18,445 to 14,776)	0.828

Values are presented as *β* coefficients (95% confidence intervals) from age- and sex-adjusted linear regression models. Confidence intervals and *p*-values were calculated using HC3 heteroscedasticity-consistent robust standard errors. *N* indicates the number of participants included in each age- and sex-adjusted regression model. Binary variables are coded as Yes = 1, No = 0. Education is coded as Junior high = 0, High school = 1, College or higher = 2. Frequency of current drinking is coded as Less than once per week = 0, At least once per week = 1, Daily = 2. CI, confidence interval. Bold values indicate statistical significance (*p* < 0.05).

Based on these findings, we constructed a multivariable linear regression model including age, sex, DDS, and migration experience to examine their independent associations with NBV (Table 3). In this model, higher dietary diversity remained significantly associated with greater NBV (*β* = 865 mm^3^ per DDS point, 95% CI 101–1,629; *p* = 0.028). Migration experience was also independently associated with higher NBV (*β* = 20,653 mm^3^, 95% CI 443–40,862; *p* = 0.046).

**Table 3:** Multivariable linear regression results for normalized brain volume (*N* = 210).

Variable	*β* (95% CI)	*p*-value
Age	−4,757 (−7,922 to −1,591)	**0.003**
Sex	16,451 (2,902 to 33,993)	**0.021**
Dietary diversity score	865 (101 to 1,629)	**0.028**
Migration	20,653 (443 to 40,862)	**0.046**

Values are presented as *β* coefficients (95% confidence intervals) from multivariable linear regression models. Confidence intervals and *p*-values were calculated using HC3 heteroscedasticity-consistent robust standard errors. Sex is coded as Female = 1, Male = 0. Migration is coded as Yes = 1, No = 0. CI = confidence interval. Statistically significant associations (*p* < 0.05) are shown in bold.

The overall pattern of results remained similar across all sensitivity analyses (Supplementary Tables S2–S4). Sensitivity analyses included (i) treating missing dietary responses as zero intake (Supplementary Table S2), (ii) using conventional standard errors instead of HC3 robust standard errors (Supplementary Table S3), and (iii) fitting a fully adjusted model that additionally included demographic, social, and clinical covariates examined in this study (Supplementary Table S4). When missing dietary responses were treated as zero intake, the association between migration experience and NBV was attenuated but remained directionally consistent (*β* = 16,684 mm^3^, *p* = 0.061), while the association between DDS and NBV remained significant (Supplementary Table S2). In the fully adjusted model, the associations of greater NBV with higher DDS (*β* = 831 mm^3^ per DDS point, 95% CI 27–1,635; *p* = 0.044) and migration experience (*β* = 28,323 mm^3^, 95% CI 4,733–51,914; *p* = 0.019) remained significant (Supplementary Table S4). All variance inflation factors were <3.

### Dietary diversity, dietary patterns, and their sociodemographic correlates

Given the observed association between dietary diversity and brain volume, we conducted additional analyses. At first, none of the individual food items showed a significant association with NBV after false discovery rate correction (Supplementary Table S5). We then performed PCA of the intake frequencies of 49 food items. The first principal component (PC1) explained 17.2% of the total variance (Supplementary Table S6). All food items had positive loadings on PC1, with particularly high loadings for vegetables, seaweed, mushrooms, potatoes, and fruits, suggesting that these food groups were major contributors to greater dietary diversity among the participants (Supplementary Table S7).

To characterize individuals with higher dietary diversity, we examined the associations between the DDS and other variables with adjustment for age and sex. In the MRI cohort, DDS was slightly higher among participants with hyperlipidemia than among those without hyperlipidemia (*p* = 0.049) (Supplementary Table S8). As a supplementary analysis, we additionally examined the associations between DDS and other variables in the entire cohort (*n* = 596) (Supplementary Table S9). Higher DDS was significantly associated with higher educational attainment (*p* < 0.001) and cohabitation (*p* < 0.001), whereas a higher Brinkman index was associated with lower DDS (*p* = 0.021).

### Additional analyses of migration characteristics

Additional analyses were performed to further characterize participants with migration experience. Among participants in the MRI study cohort for whom birthplace information was available (*n* = 18), all were born within Japan (Supplementary Table S10). In analyses of factors associated with migration experience, Brinkman index was significantly associated with migration status in the MRI cohort (Supplementary Table S11), while BMI and Brinkman index were significantly associated with migration experience in the entire cohort (Supplementary Table S12). No significant associations were observed for educational attainment, cohabitation, or dietary diversity.

## Discussion

In the present study, we aimed to identify social, lifestyle, and health-related factors associated with NBV in a rural Japanese population. Several factors were associated with NBV in the univariate analyses (Supplementary Table S1). However, the associations of smoking-related factors, alcohol-related factors, and the number of remaining teeth were attenuated after adjustment for age and sex. Previous studies have reported that these factors are associated with brain volume, brain atrophy, and the risk of dementia (1). Therefore, the lack of significant associations in the present study may reflect the relatively limited sample size or the categorization methods used in the questionnaire, rather than the absence of a true relationship.

Among the factors examined, higher dietary diversity and migration experience were independently associated with larger NBV (Tables 2, 3). Regarding dietary diversity, previous studies have likewise reported that greater dietary diversity is linked to larger brain volume, and our findings are consistent with these observations (8, 9). To further characterize dietary intake patterns, we applied PCA (Supplementary Tables S6, S7). PC1 appeared to capture the degree of dietary diversity, with particularly high loadings for vegetables, seaweed, mushrooms, potatoes, and fruits. These findings suggest that these food items contributed most strongly to dietary diversity and may be associated with greater brain volume. Previous studies have also reported that dietary patterns rich in vegetables, fruits, seaweed, and mushrooms—such as the Mediterranean diet and the traditional Japanese diet—are associated with larger brain volumes and slower progression of brain atrophy (8–10). Although the underlying mechanisms remain unclear, several explanations have been proposed, including adequate intake of vitamins and micronutrients through diverse food consumption, as well as anti-inflammatory and antioxidant effects (11).

To better understand the characteristics of individuals with higher dietary diversity, we also examined lifestyle and social factors associated with DDS (Supplementary Tables S8, S9). In the MRI cohort, Hyperlipidemia was associated with higher DDS; however, the result was close to the threshold of statistical significance and should therefore be interpreted cautiously. In contrast, analyses of the larger entire cohort demonstrated that higher dietary diversity was associated with educational attainment, cohabitation status, and Brinkman index. The associations between dietary diversity and educational attainment, cohabitation status, and smoking habits have been reported previously (12–14). These findings may provide useful information for identifying target populations when designing future interventions aimed at improving dietary diversity in this region.

In addition to DDS, migration experience emerged as a factor associated with NBV in the present study. Although numerous previous studies have examined the effects of migration on cognitive function and mental health (15–17), very few have investigated the relationship between migration experience and brain volume or brain atrophy. Sofer et al. (18) reported that immigrants to the United States who migrated after the age of 11 years had smaller intracranial volumes than native-born Americans, suggesting that migration-related experiences may influence intracranial structural development. Our findings extend this concept by suggesting that migration experience may also be associated with brain volume in later life.

The causal relationship underlying the association between migration experience and NBV remains unclear. In migration research, immigrants have often been reported to exhibit better health outcomes than native populations in countries such as the United States and Canada, a phenomenon known as the healthy immigrant effect (19). One proposed explanation is that individuals who choose to migrate tend to have higher levels of health awareness, motivation, and educational attainment than those who do not migrate. A similar mechanism may partly explain our findings. However, despite additional analyses, we found no clear or consistent differences in educational attainment, lifestyle-related diseases, or other measured characteristics that could plausibly account for the association between migration experience and NBV. Furthermore, most studies of the healthy immigrant effect have focused on migration across countries or large geographic regions. In contrast, our study was conducted within the highly localized setting of the Tango Peninsula, and the migration destinations and origins of participants were almost entirely within Japan (Supplementary Table S10). Therefore, our findings raise the possibility that migration experience may be associated with brain structure even at a much smaller geographic scale than has previously been considered. Further studies are needed to clarify the mechanisms underlying this association. In particular, more detailed analyses incorporating age at migration, reasons for migration, and duration of residence may provide important insights. In addition, investigations of individuals who migrated out of the Tango Peninsula, as well as replication studies in other regions, will be necessary to determine the generalizability of the present findings.

Several limitations of this study should be acknowledged. First, this epidemiological study was based on voluntary participation by community-dwelling residents. Therefore, selection bias may have occurred, as individuals who were more health-conscious or more motivated to participate in health-related activities may have been overrepresented. Furthermore, the primary analyses were conducted in the MRI cohort, which was selected through a screening process designed to identify individuals at potential risk of cognitive decline. Accordingly, caution is warranted when generalizing the findings to the broader population of healthy middle-aged and older adults. Indeed, compared with the entire cohort, the MRI cohort tended to show slightly higher prevalences of lifestyle-related diseases and smoking history (Table 1), which may reflect differences arising from the screening and selection process. However, the screening criteria were intentionally designed to maximize sensitivity, resulting in approximately 40% of screening participants being referred for MRI examination. In addition, the MRI cohort demonstrated generally preserved cognitive function, with a mean MMSE score of approximately 28 points. Among participants who underwent CDR assessment, all were classified as either CDR 0 or CDR 0.5. These findings suggest that the MRI cohort largely reflected the characteristics of a cognitively healthy community-dwelling older population.

Second, the accuracy of NBV measurements should be considered. MRI scans were acquired as part of routine clinical practice rather than using protocols specifically optimized for quantitative neuroimaging analyses. Consequently, some degree of measurement error in SIENAX-derived NBV estimates is inevitable. Nevertheless, all MRI examinations were performed using the same scanner and identical acquisition parameters, and NBV measurements were generated using a standardized SIENAX pipeline with identical processing parameters. In addition, visual quality control procedures were performed for all images. Because the primary objective of this study was to compare groups with different lifestyle and sociodemographic characteristics rather than to determine absolute brain volume values, we believe that the imaging methodology was appropriate for the purposes of the present study.

Third, information regarding lifestyle and sociodemographic variables was obtained through self-reported questionnaires and was therefore subject to recall bias. In addition, several potentially important factors, including occupation, physical activity, and mental health status, were not comprehensively assessed. With respect to migration experience in particular, we did not collect detailed information regarding age at migration, reasons for migration, duration of residence, or complete residential histories. These unmeasured factors may have influenced the observed associations.

Finally, because of the cross-sectional design of this study, causal relationships between brain volume and lifestyle or sociodemographic factors cannot be established. In addition, this study adopted an exploratory approach and examined a relatively large number of candidate variables potentially associated with NBV. Although this strategy was useful for identifying factors of interest, the possibility of false-positive findings due to multiple testing cannot be excluded. Therefore, the observed associations should be considered hypothesis-generating and interpreted with caution until they are confirmed in independent cohorts. Future longitudinal studies are needed to clarify the temporal relationships between these factors and brain structure, and to determine how they relate to cognitive trajectories and dementia risk over time.

In conclusion, higher dietary diversity and migration experience were independently associated with greater NBV among middle-aged and older adults in a rural Japanese population. Because of the cross-sectional design and the selected MRI cohort, causal inferences cannot be made. Longitudinal studies are needed to clarify the mechanisms underlying these associations and their implications for healthy brain aging.

## Data Availability

The raw data supporting the conclusions of this article will be made available by the authors, without undue reservation.
