# Supplementary figures and images for: Dietary diversity, migration experience, and brain volume in middle-aged and older adults in rural Japan: a cross-sectional magnetic resonance imaging study
Source: Front Public Health. 2026 Jun 26;14:1810346. doi: 10.3389/fpubh.2026.1810346 (PMC13352471; doi:10.3389/fpubh.2026.1810346)

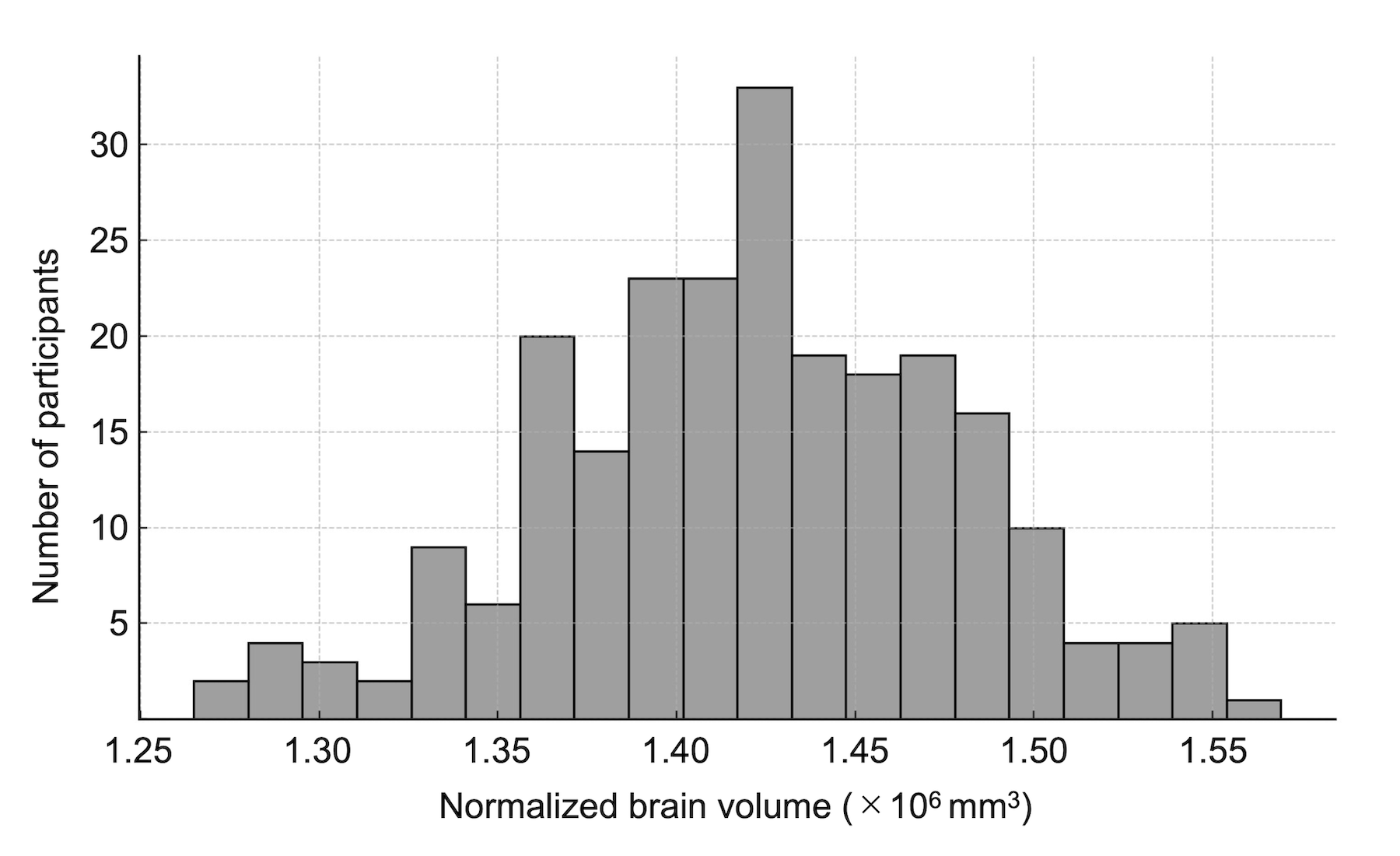

Supplement: Supplementary file 1 [file Image_1.TIFF]
