# Supplementary material for: Dietary diversity, migration experience, and brain volume in middle-aged and older adults in rural Japan: a cross-sectional magnetic resonance imaging study
Source: Front Public Health. 2026 Jun 26;14:1810346. doi: 10.3389/fpubh.2026.1810346 (PMC13352471; doi:10.3389/fpubh.2026.1810346)
Supplement: Supplementary file 2 [file Table_1.DOCX]

**Supplementary Information**

**Dietary Diversity, Migration Experience, and Brain Volume in Middle-Aged and Older Adults in Rural Japan: A Cross-Sectional Magnetic Resonance Imaging Study**

Hisashi Takahashi, Fumitoshi Niwa, Toshiya Ochiai, Satoshi Teramukai, Tomoyuki Ohara, Toshiki Mizuno, Masanori Nakagawa

**Table S1 Unadjusted linear regression results for normalized brain volume**

| **Variable** | **N** | **β (95% CI)** | **p-value** |
| --- | --- | --- | --- |
| Sex | 235 | 23,519 (8,963 to 38,074) | **0.002** |
| Dietary diversity score | 210 | 1,038 (337 to 1,739) | **0.004** |
| Smoking history | 235 | -21,270 (-35,883 to -6,657) | **0.005** |
| Age | 235 | -4,283 (-7,376 to -1,191) | **0.007** |
| Brinkman index | 230 | -23.7 (-41.5 to -6.0) | **0.009** |
| Migration | 235 | 20,439 (2,238 to 38,641) | **0.028** |
| Number of remaining teeth | 233 | 1,021 (28 to 2,013) | **0.044** |
| Current smoker | 235 | -22,814 (-48,098 to 2,470) | 0.077 |
| Frequency of current drinking | 229 | -8,264 (-17,633 to 1,105) | 0.084 |
| Alcohol history | 234 | -12,695 (-27,794 to 2,405) | 0.099 |
| Diabetes | 235 | -22,603 (-49,646 to 4,440) | 0.101 |
| Hyperlipidemia | 235 | 13,591 (-3,947 to 31,129) | 0.128 |
| Mini-Mental State Examination score | 235 | -2,670 (-6,372 to 1,031) | 0.157 |
| Cohabitant | 235 | 6,383 (-4,594 to 17,360) | 0.253 |
| Hypertension | 235 | -7,005 (-23,128 to 9,118) | 0.393 |
| Education | 211 | 5,147 (-6,766 to 17,060) | 0.395 |
| Body mass index | 235 | 555 (-1,627 to 2,737) | 0.617 |
| Currently married | 215 | -4,626 (-24,421 to 15,169) | 0.646 |

Note. Values are presented as β coefficients (95% confidence intervals) from univariate linear regression models. Confidence intervals and p-values were calculated using HC3 heteroscedasticity-consistent robust standard errors. N indicates the number of participants included in each regression model. Sex is coded as Female = 1, Male = 0. Other binary variables are coded as Yes = 1, No = 0. Education is coded as Junior high = 0, High school = 1, College or higher = 2. Frequency of current drinking is coded as Less than once per week = 0, At least once per week = 1, Daily = 2. CI = confidence interval. Bold values indicate statistical significance (p < 0.05).

**Table S2. Sensitivity analysis treating missing dietary responses as zero intake (N = 235)**

| **Variable** | **β (95% CI)** | **p-value** |
| --- | --- | --- |
| Age | -4,276 (-7,208 to -1,343) | **0.005** |
| Sex | 16,451 (1,208 to 31,694) | **0.035** |
| Dietary diversity score | 878 (125 to 1,630) | **0.023** |
| Migration | 16,684 (-120 to 34,569) | 0.061 |

Note. Values are presented as β coefficients (95% confidence intervals) from multivariable linear regression models using HC3 heteroscedasticity-consistent robust standard errors. As a sensitivity analysis, missing dietary diversity score values were treated as zero. Sex is coded as Female = 1, Male = 0. Migration is coded as Yes = 1, No = 0. CI = confidence interval. Statistically significant associations (p < 0.05) are shown in bold.

**Table S3. Sensitivity analysis using conventional ordinary least squares standard errors for normalized brain volume (N = 210)**

| **Variable** | **β (95% CI)** | **p-value** |
| --- | --- | --- |
| Age | -4,756 (-8,062 to -1,451) | **0.005** |
| Sex | 18,448 (3,158 to 33,738) | **0.018** |
| Dietary diversity score | 865 (120 to 1,609) | **0.023** |
| Migration | 20,653 (1,760 to 39,545) | **0.032** |

Note. Values are presented as β coefficients (95% confidence intervals) from multivariable linear regression models using ordinary least squares (OLS) standard errors as a sensitivity analysis. Sex is coded as Female = 1, Male = 0. Migration is coded as Yes = 1, No = 0. CI = confidence interval. Statistically significant associations (p < 0.05) are shown in bold.

**Table S4. Fully adjusted multivariable model (N = 185)**

| **Variable** | **β (95% CI)** | **p-value** |
| --- | --- | --- |
| Migration | 28,323 (4,733 to 51,914) | **0.019** |
| Dietary diversity score | 831 (27 to 1,635) | **0.044** |
| Diabetes | -24,113 (-51,690 to 3,463) | 0.088 |
| Number of remaining teeth | 714 (-424 to 1,853) | 0.221 |
| Age | -2,178 (-5,759 to 1,403) | 0.235 |
| Cohabitant | 742 (-6,805 to 21,708) | 0.307 |
| Sex | -14,713 (-51,126 to 21,700) | 0.43 |
| Education | 5,302 (-831 to 18,916) | 0.446 |
| Body mass index | 1,100 (-1,844 to 4,044) | 0.465 |
| Hypertension | 5,829 (-14,372 to 26,031) | 0.572 |
| Hyperlipidemia | 4,986 (-18,128 to 28,101) | 0.673 |
| Currently married | -4,049 (-25,748 to 17,651) | 0.715 |
| Smoking history | -2,900 (-39,458 to 33,657) | 0.876 |
| Alcohol history | -167 (-21,128 to 20,795) | 0.987 |

Note. Values are presented as β coefficients (95% confidence intervals) from a fully adjusted multivariable linear regression model using HC3 heteroscedasticity-consistent robust standard errors. Sex is coded as Female = 1, Male = 0. Other binary variables are coded as Yes = 1, No = 0. Education is coded as Junior high = 0, High school = 1, College or higher = 2. CI = confidence interval. Statistically significant associations (p < 0.05) are shown in bold.

**Table S5. Age- and sex-adjusted partial correlations between food intake and brain volume**

| **Food** | **N** | **Partial r** | **p-value** | **q-value (FDR)** | **FDR significant** |
| --- | --- | --- | --- | --- | --- |
| Natto/soybeans | 234 | 0.157 | 0.0163 | 0.356 | FALSE |
| Cheese | 235 | 0.141 | 0.0303 | 0.356 | FALSE |
| Beef | 235 | 0.141 | 0.0311 | 0.356 | FALSE |
| Ham/processed meat | 235 | 0.136 | 0.0376 | 0.356 | FALSE |
| Dark green vegetables | 235 | 0.133 | 0.0417 | 0.356 | FALSE |
| Fried foods | 232 | 0.128 | 0.0521 | 0.356 | FALSE |
| Pork | 235 | 0.126 | 0.0539 | 0.356 | FALSE |
| Butter | 234 | 0.121 | 0.0656 | 0.356 | FALSE |
| Tsukudani (preserved foods) | 234 | 0.114 | 0.0822 | 0.356 | FALSE |
| Nuts | 235 | 0.113 | 0.0825 | 0.356 | FALSE |
| Tofu | 235 | 0.109 | 0.0957 | 0.356 | FALSE |
| Green tea | 235 | 0.108 | 0.0972 | 0.356 | FALSE |
| Light-colored vegetables | 234 | 0.107 | 0.1022 | 0.356 | FALSE |
| Fish cakes | 235 | 0.106 | 0.1066 | 0.356 | FALSE |
| Eggs | 235 | 0.105 | 0.109 | 0.356 | FALSE |
| Cabbage | 232 | 0.087 | 0.1859 | 0.5179 | FALSE |
| Stir-fried vegetables | 235 | 0.086 | 0.1869 | 0.5179 | FALSE |
| Liver | 235 | 0.086 | 0.1903 | 0.5179 | FALSE |
| Yogurt | 235 | 0.08 | 0.2197 | 0.5667 | FALSE |
| Other fruits | 235 | 0.072 | 0.2743 | 0.6721 | FALSE |
| Deep-fried tofu | 235 | 0.067 | 0.3041 | 0.6878 | FALSE |
| Black tea | 234 | 0.067 | 0.3088 | 0.6878 | FALSE |
| Seaweed | 235 | 0.058 | 0.3767 | 0.7723 | FALSE |
| Milk | 235 | 0.054 | 0.4075 | 0.7723 | FALSE |
| Miso soup | 235 | 0.054 | 0.4112 | 0.7723 | FALSE |
| Wild vegetables | 234 | 0.052 | 0.4267 | 0.7723 | FALSE |
| Pickles | 235 | 0.05 | 0.4413 | 0.7723 | FALSE |
| Tomatoes | 232 | 0.051 | 0.4413 | 0.7723 | FALSE |
| Potatoes | 235 | 0.049 | 0.458 | 0.7739 | FALSE |
| Blue-back fish | 234 | 0.04 | 0.5388 | 0.88 | FALSE |
| Japanese-style sweets | 235 | 0.036 | 0.5829 | 0.8935 | FALSE |
| Shellfish | 235 | 0.033 | 0.6165 | 0.8935 | FALSE |
| Small fish | 235 | 0.031 | 0.6404 | 0.8935 | FALSE |
| Carrots/pumpkin | 235 | 0.027 | 0.686 | 0.8935 | FALSE |
| Fish (general) | 235 | -0.026 | 0.6934 | 0.8935 | FALSE |
| Margarine | 234 | -0.024 | 0.7155 | 0.8935 | FALSE |
| Carbonated drinks | 235 | 0.022 | 0.7322 | 0.8935 | FALSE |
| Canned tuna | 232 | 0.023 | 0.7326 | 0.8935 | FALSE |
| Dried/salted fish | 233 | -0.021 | 0.7519 | 0.8935 | FALSE |
| Coffee | 234 | 0.02 | 0.7626 | 0.8935 | FALSE |
| Chinese tea | 233 | 0.02 | 0.7627 | 0.8935 | FALSE |
| Western-style sweets | 235 | 0.02 | 0.7658 | 0.8935 | FALSE |
| Citrus fruits | 233 | 0.017 | 0.8005 | 0.9091 | FALSE |
| Fruit juice | 234 | 0.015 | 0.8163 | 0.9091 | FALSE |
| Roe | 234 | -0.009 | 0.8872 | 0.966 | FALSE |
| Chicken | 235 | -0.007 | 0.917 | 0.9767 | FALSE |
| Mushrooms | 235 | 0.004 | 0.9566 | 0.9767 | FALSE |
| Squid | 234 | -0.004 | 0.9567 | 0.9767 | FALSE |
| Leafy greens | 235 | -0.001 | 0.9836 | 0.9836 | FALSE |

Note. Partial correlations were calculated between each food item and brain volume, adjusted for age and sex. p-values were corrected for multiple comparisons using the Benjamini–Hochberg false discovery rate (FDR). Significant results after FDR correction are indicated in the column 'FDR significant'.

**Table S6. Variance explained by the first three principal components (PC1–PC3) of dietary intake**

| **Principal Component** | **Explained Variance Ratio (%)** | **Cumulative Variance (%)** |
| --- | --- | --- |
| PC1 | 17.22 | 17.22 |
| PC2 | 5.68 | 22.91 |
| PC3 | 5.08 | 27.98 |

Notes. Principal components (PC1–PC3) were derived from principal component analysis (PCA) using the intake frequencies of 49 food items. PCA was conducted among participants with complete data on all 49 food items (n = 210). Explained variance ratio indicates the proportion of variance in dietary intake explained by each component, and cumulative variance ratio represents the cumulative variance explained up to that component.

**Table S7. Factor loadings of all 49 food items on PC1**

| **Food Item** | **N** | **Factor Loading (PC1)** |
| --- | --- | --- |
| Leafy greens | 235 | 0.225 |
| Light-colored vegetables | 234 | 0.22 |
| Carrots/pumpkin | 235 | 0.218 |
| Seaweed | 235 | 0.216 |
| Deep-fried tofu (Ganmodoki etc) | 235 | 0.209 |
| Dark green vegetables | 235 | 0.207 |
| Mushrooms | 235 | 0.202 |
| Potatoes | 235 | 0.202 |
| Citrus fruits | 233 | 0.197 |
| Other fruits | 235 | 0.186 |
| Small fish | 235 | 0.18 |
| Tomatoes | 232 | 0.178 |
| Japanese-style sweets | 235 | 0.176 |
| Stir-fried vegetables | 235 | 0.176 |
| Tofu | 235 | 0.174 |
| Fish cakes | 235 | 0.163 |
| Natto/soybeans | 234 | 0.162 |
| Western-style sweets | 235 | 0.159 |
| Cabbage | 232 | 0.148 |
| Nuts | 235 | 0.145 |
| Squid | 235 | 0.139 |
| Wild vegetables | 234 | 0.139 |
| Tsukudani (preserved foods) | 234 | 0.136 |
| Roe | 234 | 0.133 |
| Black tea | 234 | 0.13 |
| Fish (general) | 235 | 0.13 |
| Dried/salted fish | 235 | 0.127 |
| Pork | 235 | 0.119 |
| Shellfish | 235 | 0.117 |
| Beef | 235 | 0.117 |
| Chicken | 235 | 0.102 |
| Fruit juice | 234 | 0.101 |
| Pickles | 235 | 0.1 |
| Cheese | 235 | 0.1 |
| Milk | 235 | 0.099 |
| Blue-back fish | 234 | 0.098 |
| Miso soup | 235 | 0.094 |
| Butter | 234 | 0.09 |
| Yogurt | 235 | 0.089 |
| Canned tuna | 232 | 0.084 |
| Liver | 235 | 0.078 |
| Ham/processed meat | 235 | 0.075 |
| Fried foods | 232 | 0.069 |
| Green tea | 235 | 0.068 |
| Carbonated drinks | 235 | 0.066 |
| Eggs | 235 | 0.05 |
| Chinese tea | 233 | 0.027 |
| Margarine | 234 | 0.025 |
| Coffee | 234 | 0.02 |

Notes. Factor loadings were derived from principal component analysis (PCA) using the intake frequencies of 49 food items. Unless otherwise noted, n = 235. Factor loading values indicate the strength and direction of association between each food item and the first principal component (PC1). All food items showed positive loadings on PC1, indicating that PC1 represents a general tendency to consume a wide variety of foods, consistent with greater dietary diversity. Higher factor loadings denote stronger contributions of individual food items to PC1.

**Table S8. Age- and sex-adjusted linear regression results for the dietary diversity score for the MRI cohort**

| **Variable** | **N** | **β (95% CI)** | **p-value** |
| --- | --- | --- | --- |
| Hyperlipidemia | 210 | 3.27 (0.02 to 6.52) | **0.049** |
| Body mass index | 210 | 0.36 (-0.10 to 0.82) | 0.125 |
| Married | 193 | -2.60 (-6.63 to 1.42) | 0.205 |
| Smoking history | 210 | -3.20 (-8.27 to 1.87) | 0.216 |
| Mini-Mental State Examination score | 210 | -0.65 (-1.76 to 0.46) | 0.25 |
| Migration | 210 | 1.89 (-1.88 to 5.65) | 0.326 |
| Frequency of current drinking | 205 | -1.00 (-3.04 to 1.05) | 0.33 |
| Hypertension | 210 | -1.37 (-4.86 to 2.12) | 0.441 |
| Current smoker | 210 | -1.93 (-7.31 to 3.46) | 0.483 |
| Cohabitant | 210 | -0.30 (-1.18 to 0.58) | 0.502 |
| Diabetes | 210 | 1.98 (-4.90 to 8.86) | 0.573 |
| Education | 189 | -0.58 (-3.25 to 2.09) | 0.669 |
| Alcohol history | 209 | -0.69 (-4.01 to 2.64) | 0.686 |
| Number of remaining teeth | 208 | 0.04 (-0.18 to 0.27) | 0.71 |
| Brinkman index | 205 | -0.0012 (-0.0084 to 0.0060) | 0.748 |

Notes. Values are presented as β coefficients (95% confidence intervals) from age- and sex-adjusted linear regression models using HC3 heteroscedasticity-consistent robust standard errors. N indicates the number of participants included in each age- and sex-adjusted regression model. Binary variables are coded as Yes = 1, No = 0. Education is coded as Junior high = 0, High school = 1, College or higher = 2. Frequency of current drinking is coded as Less than once per week = 0, At least once per week = 1, Daily = 2. CI = confidence interval. Bold values indicate statistical significance (p < 0.05).

**Table S9. Age- and sex-adjusted linear regression results for the dietary diversity score for the entire cohort.**

| **Variable** | **N** | **β (95% CI)** | **p-value** |
| --- | --- | --- | --- |
| Education | 482 | 2.71 (1.44 to 3.99) | **<0.001** |
| Cohabitant | 532 | 4.56 (2.00 to 7.11) | **<0.001** |
| Brinkman index | 526 | -0.0027 (-0.0050 to -0.0004) | **0.021** |
| Currently married | 505 | 2.04 (-0.05 to 4.14) | 0.056 |
| Hypertension | 531 | -1.46 (-3.12 to 0.19) | 0.083 |
| Migration | 533 | 1.69 (-0.35 to 3.73) | 0.105 |
| Current smoker | 533 | -2.34 (-5.46 to 0.77) | 0.141 |
| Smoking history | 533 | -1.47 (-3.74 to 0.80) | 0.205 |
| Frequency of current drinking | 532 | 0.46 (-0.49 to 1.41) | 0.345 |
| Hyperlipidemia | 532 | 0.68 (-1.10 to 2.45) | 0.455 |
| Body mass index | 533 | 0.08 (-0.16 to 0.32) | 0.514 |
| Mini-mental examination score | 532 | 0.16 (-0.34 to 0.67) | 0.521 |
| Number of remaining teeth | 520 | 0.03 (-0.08 to 0.15) | 0.602 |
| Diabetes | 533 | -0.42 (-3.65 to 2.80) | 0.797 |
| Alcohol history | 532 | -0.14 (-1.75 to 1.46) | 0.862 |

Notes. Values are presented as β coefficients (95% confidence intervals) from age- and sex-adjusted linear regression models using HC3 heteroscedasticity-consistent robust standard errors. N indicates the number of participants included in each age- and sex-adjusted regression model. Binary variables are coded as Yes = 1, No = 0. Education is coded as Junior high = 0, High school = 1, College or higher = 2. Frequency of current drinking is coded as Less than once per week = 0, At least once per week = 1, Daily = 2. CI = confidence interval. Bold values indicate statistical significance (p < 0.05).

**Table S10. Birthplace distribution among participants with migration experience in the MRI cohort**

| **Birthplace** | **Number of participants** |
| --- | --- |
| Within Kyoto prefecture | 4 |
| Outside Kyoto prefecture | 14 |

Note. Among participants with migration experience who provided information on birthplace (n = 18), 4 were born elsewhere within Kyoto Prefecture and 14 were born in other prefectures of Japan, indicating that migration in this cohort primarily reflected domestic relocation rather than international migration.

**Table S11. Age- and sex-adjusted logistic regression analyses of factors associated with migration status in the MRI cohort**

| **Variable** | **N** | **OR (95% CI)** | **p value** |
| --- | --- | --- | --- |
| Brinkman index | 230 | 1.001 (1.000 to 1.002) | **0.009** |
| Body mass index | 235 | 1.093 (0.989 to 1.208) | 0.081 |
| Education | 211 | 0.647 (0.382 to 1.096) | 0.105 |
| Current smoker | 235 | 2.167 (0.711 to 6.607) | 0.174 |
| Cohabitant | 235 | 0.548 (0.224 to 1.340) | 0.187 |
| Frequency of current drinking | 229 | 0.737 (0.455 to 1.196) | 0.217 |
| Dietary diversity score | 210 | 1.018 (0.985 to 1.053) | 0.288 |
| Diabetes | 235 | 1.621 (0.538 to 4.889) | 0.391 |
| Mini-mental state examination score | 235 | 0.937 (0.802 to 1.094) | 0.411 |
| Alcohol history | 234 | 1.298 (0.629 to 2.677) | 0.48 |
| Hyperlipidemia | 235 | 1.242 (0.598 to 2.582) | 0.561 |
| Hypertension | 235 | 0.878 (0.425 to 1.814) | 0.726 |
| Number of remaining teeth | 233 | 0.997 (0.954 to 1.041) | 0.887 |
| Smoking history | 235 | 1.035 (0.468 to 2.286) | 0.933 |
| Currently married | 215 | 0.990 (0.418 to 2.342) | 0.981 |

Note. Odds ratios (ORs), 95% confidence intervals (CIs), and p values were obtained from separate logistic regression models with migration status (yes/no) as the dependent variable. Each model included age, sex, and the variable of interest. HC3 heteroscedasticity-consistent robust standard errors were used. Binary variables are coded as Yes = 1, No = 0. Education is coded as Junior high = 0, High school = 1, College or higher = 2. Frequency of current drinking is coded as Less than once per week = 0, At least once per week = 1, Daily = 2. Sample size varied across analyses because of missing data. Statistically significant associations (p < 0.05) are shown in bold.

**Table S12. Age- and sex-adjusted logistic regression analyses of factors associated with migration status in the entire cohort**

| **Variable** | **N** | **OR (95% CI)** | **p-value** |
| --- | --- | --- | --- |
| Brinkman index | 587 | 1.001 (1.000 to 1.001) | **0.007** |
| Body mass index | 596 | 1.072 (1.012 to 1.136) | **0.017** |
| Alcohol history | 595 | 1.569 (0.992 to 2.483) | 0.054 |
| Dietary diversity score | 533 | 1.023 (0.997 to 1.051) | 0.089 |
| Number of remaining teeth | 583 | 1.020 (0.989 to 1.053) | 0.211 |
| Smoking history | 596 | 1.455 (0.799 to 2.650) | 0.22 |
| Hypertension | 594 | 0.755 (0.465 to 1.226) | 0.256 |
| Cohabitant | 595 | 0.694 (0.348 to 1.386) | 0.301 |
| Hyperlipidemia | 595 | 0.826 (0.499 to 1.367) | 0.457 |
| Current smoker | 596 | 1.293 (0.559 to 2.989) | 0.548 |
| Diabetes | 596 | 1.250 (0.568 to 2.749) | 0.579 |
| Frequency of current drinking | 595 | 1.077 (0.826 to 1.405) | 0.585 |
| Currently married | 564 | 1.116 (0.631 to 1.977) | 0.706 |
| Mini-mental examination score | 595 | 1.017 (0.902 to 1.147) | 0.785 |
| Education | 538 | 1.030 (0.722 to 1.470) | 0.869 |

Note. Odds ratios (OR), 95% confidence intervals (CI), and p values were obtained from separate logistic regression models with migration status (yes/no) as the dependent variable. Each model included age, sex, and the variable of interest. HC3 heteroscedasticity-consistent robust standard errors were used. Binary variables are coded as Yes = 1, No = 0. Education is coded as Junior high = 0, High school = 1, College or higher = 2. Frequency of current drinking is coded as Less than once per week = 0, At least once per week = 1, Daily = 2. Sample size varied across analyses because of missing data. Statistically significant associations (p < 0.05) are shown in bold.

**Supplementary Figure Legends**

Supplementary Figure S1. Distribution of normalized brain volume (NBV)

Histogram showing the distribution of NBV among 235 participants after outlier removal.
